# Supplementary material for: OpenCap: Human movement dynamics from smartphone videos
Source: PLoS Comput Biol. 2023 Oct 19;19(10):e1011462. doi: 10.1371/journal.pcbi.1011462 (PMC10586693; doi:10.1371/journal.pcbi.1011462)
Supplement: Attachment — Submitted filename: ResponseToReviewers.pdf [file pcbi.1011462.s007.pdf]

Response to Reviewers

Article: OpenCap: human movement dynamics from smartphone videos

Manuscript number: PCOMPBIOL-D-23-00154

Response

We thank the reviewers for the constructive feedback. Revising the manuscript based on these comments has improved its quality and clarity.

General comments:

1) We changed the title of the manuscript from "OpenCap: motor control and musculoskeletal forces from smartphone videos" to "OpenCap: human movement dynamics from smartphone videos." The new title more appropriately describes the breadth of OpenCap's capabilities.

2) After the first submission of the manuscript, two authors started a company to make this open-source software accessible to commercial users who are not performing academic research. This does not change the platform's free availability to the research community, which we originally described in the manuscript: the open-source code is actively developed for research and the cloud-based OpenCap platform is hosted by Stanford University and is freely available to anyone conducting academic research. The competing interests statement was updated accordingly:

   "Scott Uhlrich and Antoine Falisse are co-founders of Model Health, Inc., which supports the non-academic, commercial use of the open-source software described here. OpenCap, the cloud-deployed academic version of this open-source software is hosted at Stanford University and will remain freely available to the academic research community for the foreseeable future. No other authors have competing interests to declare."

Reviewer #1: This is a well-written paper describing an innovative system with obvious applications in the clinic and in the "wild".

Please provide context for error values given in the validation section (lines 156-158, etc). For instance, do the MAE errors have the potential to affect clinical decision making?

We compare OpenCap's kinematic and kinetic errors to other portable motion capture systems (inertial measurement units and eight-camera markerless motion capture systems) in the

Discussion (lines 383–410). To further contextualize these errors, we added a comparison of the kinematic differences we report between OpenCap and skin-mounted marker-based motion capture and the documented errors between skin-mounted and bone pin–mounted marker-based motion capture (lines 388–396). For the knee angle during walking, OpenCap's errors (4.2° RMSE) are smaller than the errors that can arise from skin motion artifact (8° RMSE) in marker-based motion capture. Additionally, the differences between OpenCap and marker-based motion capture are similar to the differences observed for 17-IMU and eight-camera markerless motion capture systems, demonstrating that it may be challenging to achieve greater concordance with marker-based motion capture due to the underlying errors in the marker-based approach that we used as ground truth. We also added this point to the Discussion (lines 388–396).

The accuracy required to inform clinical decision making varies by population, activity, metric, decision, etc., so we cannot make general claims about how our joint angle, joint torque, and ground force errors impact clinical decisions. For this reason, we investigated if OpenCap could estimate several scalar metrics that could inform clinical decisions or research findings across several activities (Figures 3–6). For metrics with established clinically meaningful accuracy thresholds (e.g., the knee adduction moment), we compared OpenCap's accuracy to these thresholds. In response to the reviewer's important point, we added a sentence to the discussion encouraging researchers to validate that OpenCap is sufficiently accurate for their specific use case, when extending it to populations, activities, and metrics, that were not explored in this paper (lines 349–352).

Please discuss scatter in the predictive accuracy shown (e.g., Figures 3 and 4). Do errors in the accuracy of predicting peak MCF or knee extension moment have the potential to affect clinical decision making? Are there any indications as to the cause of larger errors in specific individuals / tests / motions?

We appreciate the importance of contextualizing the predictive accuracy of scalar metrics shown in Figures 3 and 4. Unfortunately, clinically relevant thresholds are not always available for the measures we examined since these values have traditionally been so resource-intensive to estimate. Thus, we compare as much as possible to clinical thresholds and complement with comparisons to thresholds applicable to research. OpenCap enables the large-scale biomechanics and outcomes studies necessary to create clinically relevant thresholds, which we hope will improve the clinical utility of estimating movement dynamics. We have added a discussion of this point in lines 425–432.

In Figure 3, we compare the MAE of the knee adduction moment (KAM) to between-group differences in KAM between individuals who do and do not progress with osteoarthritis—our errors are below these thresholds. This is possible due to a large body of longitudinal studies relating the KAM to knee osteoarthritis progression. There is only one study relating medial contact force (MCF) to osteoarthritis progression [1], and it does not report values that could be used as a threshold to contextualize the errors in our study. For this reason, we only report and contextualize the MAE for the value of the peak KAM.

We can contextualize how correctly predicting the gait modification–induced *changes* in peak KAM and peak MCF influences clinical decision making. For all 10 participants, OpenCap correctly identified an increase or reduction in both KAM and MCF. We describe this result in line 182–184. We more clearly depicted the participants who increased and reduced loading in Figure 3B and its caption to show that OpenCap predicts the same direction of change as motion capture for individual participants.

For Figure 4, we are not aware of values in the literature that can be used to create clinically meaningful accuracy thresholds for changes in knee extension moment when rising from a chair. To contextualize the error without such a threshold, we added a comparison between OpenCap's error (MAE=5.5 Nm) and the annual loss in dynamic strength due to age (0.93Nm per year [2]), suggesting that our errors are similar to the average strength reductions that occur over a six-year period (lines 223–226). To further contextualize the impact of OpenCap's errors on research studies, we compare statistical power for detecting between-condition changes in joint moments when using the outputs of motion capture and OpenCap (lines 177–182 and 220–223).

A comprehensive sensitivity analysis of kinetic errors in Figures 3 and 4 is beyond the scope of this study. Many subject- and activity-specific factors (e.g., mass distribution, inertial parameters, soft tissue motion, shoe properties, etc.) could create errors in both the OpenCap and marker-based motion capture pipelines. Many of these error sources we cannot quantify, and the contribution of even simple errors (e.g., joint angle errors) have a nonlinear relationship to kinetics. An in-depth commentary of the source of these errors would be speculative, but we can make some observations about the nature of the errors:

1) For both knee loading measures, we correctly predict the direction of change in knee loading induced by a gait modification for all participants (Figure 3B). This suggests that for an individual, the error magnitude did not substantially change between different walking patterns. This is mentioned in the Figure 3 caption and in the manuscript.
2) We added a y=x line to Figure 4B, showing that the errors in peak knee extension moment increase as the magnitude of the moment increases. We added discussion of this potential source of error, as well as ideas for mitigating it in the future, in Appendix S1:

   Added comment about error source in Appendix S1, referenced in line 226 of manuscript:

   "We aimed to keep our problem formulation as general as possible, but more accurate solutions can likely be obtained by further tailoring the formulation to specific tasks. For example, minimizing muscle effort is a good term for regularizing muscle activations during walking; however, humans do not minimize effort during all activities. This may explain why OpenCap underpredicted the knee extension moment (i.e., quadriceps effort) during sit-to-stand (Fig 4B) more as the magnitude of the moment increased. A more task-specific cost function may improve accuracy for tasks, like the sit-to-stand,

where additional terms, like maintaining stability or maximizing speed, may compete with muscle effort as the movement objective."

Please discuss error relative to the capture volume. How does error relate to depth of field or position of the individual relative to the iPhone cameras or within the capture volume?

We did not quantify how depth of field and position of the subject in the capture volume affect OpenCap's results. We added a section to provide guidance on the size of the capture volume and sources of error at different locations in the volume (new "Practical considerations" section, lines 576–615). Specifically, pose estimation is most accurate 2–10m from the camera, and the 3D markers will be most accurate near where the checkerboard was placed during calibration. Furthermore, the volume can be maximized by ensuring the participant enters and exits the field of view of both cameras at the same location in the volume. The participant's position in the camera field of view likely does not affect accuracy since we undistort images based on the intrinsic parameters computed for all iOS devices after 2018.

Please discuss repeatability of measurements.

We did not quantify the repeatability of measurements; however, we anticipate our results to follow the conclusions from previous research on repeatability of measurements from markerless motion capture. Kanko et al. (2021) reported inter-trial variability, inter-session variability, and the ratio between them [3]. Although their study focused on gait kinematics only, they reported inter-trial variability of on average 2.5°, inter-session variability of on average 2.8°, and a variability ratio of 1.1 on average (this indicates that the inter-session variability increased the total variability by 10% compared to the inter-trial variability only). The results are similar or lower than values reported for marker-based studies, suggesting that gait kinematics can be reliably measured using markerless motion capture. Given the common underlying methods, we believe OpenCap's repeatability should follow similar trends for gait and other activities. We added a reference to the Kanko et al. repeatability study [3] and acknowledge the lack of a repeatability analysis in the limitations paragraph of the Discussion (lines 432–437).

Please briefly discuss practical guidance/requirements (e.g., clothing, lighting, other) for capturing iPhone video in the clinic or in the wild.

Thank you for this helpful suggestion. We added a section, "Practical considerations," to the Methods (lines: 576–615). In this section, we also refer users to visit our website, opencap.ai, where we provide tutorials and guidance for collecting high-quality data. We will continue to keep this website up to date after publication of the manuscript.

Reviewer #2: I would like to congratulate the authors of "OpenCap: motor control and musculoskeletal forces from smartphone videos" on an important and transformative contribution to the field. In this manuscript, the authors present, validate, and test OpenCap, an open-source platform for computing kinematics and kinetics of human

movement using videos captured from smartphones. This is a transformative contribution to the field as it provides a tool for low-cost analysis of human movement, that can be easily administered in a clinical setting or practical environment, and can quickly output relevant metrics from a large cohort. The authors well validate the platform and test its utility for screening for disease risk, evaluating interventions, and informing rehabilitation. The manuscript is well written, all figures are of the highest quality, and all data is openly shared, with additional detailed descriptions in the supplemental material and elsewhere. I only have minor comments, which are provided below.

Minor comments:
Line 7: consider specifying “two or more smartphones” or “a minimum of two” instead of simply two

We adjusted this sentence following the reviewer’s suggestion.

In lines 527-536 the authors describe how OpenCap can estimate dynamics using muscle driven tracking simulations of joint kinematics. However, it is unclear to me how the ground reaction forces are estimated and I would appreciate clarification on this point. I have referred to Appendix 1: Optimal control formulations to seek an answer. Potentially the GRFs were allowed to vary in the muscle driven tracking simulation and then computed based on the simulation that minimized the cost function specified in line 535? Given this statement (from OpenSim documentation) “Note that you must measure or model all external forces acting on a subject during the motion to calculate accurate muscle forces” it seems to me the GRFs must have been modeled somehow as they were not measured for the OpenCap condition.

Ground reaction forces are estimated as part of the muscle-driven tracking simulations of joint kinematics (Methods: Physics-based modeling and simulation). To estimate these forces, six contact spheres are affixed to each foot of the musculoskeletal model, and a ground contact plane is defined. The contact model employed is a smooth approximation of a compliant Hunt-Crossley foot-ground contact. When solving the optimal control problem underlying the muscle-driven tracking simulation, ground forces and torques are produced between each contact sphere and the ground as a function of the states of the model (i.e., joint coordinate values and speeds). These forces and torques are then input to the rigid body dynamic equations describing the motion of the skeleton.

The model states are the design variables in the optimal control problem, changing until the problem reaches an optimal solution. As the contact models generate forces and torques based on these model states, they too will fluctuate throughout the optimal control process. However, unlike the model states, the forces and torques are not design variables within the problem. Once an optimal solution is attained, the equations governing the skeleton's dynamics (i.e., the equations of motion) are satisfied, resulting in a dynamically consistent solution where no pelvis residuals are required.

As described in the OpenSim documentation, external forces must be measured or modeled. As explained above, ground reaction forces and torques are thus modeled in the case of OpenCap. We adjusted the manuscript to make this methodological point clearer (lines 535–537).

Please comment on whether users can select a musculoskeletal model for use with OpenCap or whether OpenCap must use the model from Lai et al. with modified hip abductor muscle paths.

The default model supported by OpenCap is the model from Lai et al. with modified hip abductor muscle paths. This is the model we used to compute the results reported in the paper. We recently added support for a modified version of that model that incorporates a six degree-of-freedom shoulder complex joint that follows conventions from the ISB [4]. We are planning to support other models in the future based on the needs of the community. We referenced the availability of multiple musculoskeletal models in the Methods (lines 612–615). We also share the source code of OpenCap (https://github.com/stanfordnmbl/opencap-core), which researchers can use to reprocess their data locally with a different musculoskeletal model.

Lines 685-687: It is my understanding that the OpenCap muscle driven dynamic simulation is a forward dynamics approach, such that muscle activation dynamics are modeled. For the first application in assessing peak knee adduction moment and peak medial knee contact force during walking, OpenCap dynamic simulation is compared to MoCap static optimization (using OpenSim Static Optimization utility). Can the authors please comment on the choice of static optimization (which cannot account for muscle activation dynamics) here when a more similar comparison may have been to Mocap Computed Muscle Control driven forward simulation, which does account for muscle activation dynamics?

It is correct that the muscle-driven dynamic simulations used in OpenCap take muscle activation dynamics into account, whereas static optimization (SO) does not. We chose to use SO, compared to computed muscle control (CMC), because SO is commonly used for estimating knee contact force and several studies have validated these estimates against knee contact forces from instrumented knee implants (e.g., [5]). Fewer studies validate the use of CMC for estimating knee contact forces. We added this explanation to the Methods (lines 755–757).

Despite being more commonly used in the knee loading literature, SO has more differences compared to OpenCap's muscle-driven dynamic simulations than CMC does, so our reported errors between OpenCap and SO may be larger than those between OpenCap and CMC; however, we found it most important to compare OpenCap to the commonly used and well-validated approach for our metrics of interest (SO). Furthermore, the tasks for which we used SO were not highly dynamic (walking and squats), thus the differences in muscle forces computed by CMC and SO are likely negligible; previous work shows that patterns of muscle force estimates from SO and CMC are similar for walking and running [6]. Additionally, using CMC requires considerable effort to find the right parameters (e.g., tracking gains and

coordinate weights) and obtain meaningful results. Given the aforementioned reasons, using CMC to simulate the Mocap data was not superior to using SO.

Lines 716-718 Same question as above regarding muscle driven tracking optimization versus static optimization but now in the context of squatting.

See answer above.

Line 381: Typo. Correct to read "inertial-measurement-unit-based" please.

We corrected all occurrences of this phrase to read “inertial measurement unit–based.”

References

1. Brisson NM, Gatti AA, Damm P, Duda GN, Maly MR. Association of Machine Learning-Based Predictions of Medial Knee Contact Force With Cartilage Loss Over 2.5 Years in Knee Osteoarthritis. Arthritis Rheumatol. 2021;73: 1638–1645. doi:10.1002/art.41735

2. Larsson L, Karlsson J. Isometric and dynamic endurance as a function of age and skeletal muscle characteristics. Acta Physiol Scand. 1978;104: 129–136. doi:10.1111/j.1748-1716.1978.tb06259.x

3. Kanko RM, Laende E, Selbie WS, Deluzio KJ. Inter-session repeatability of markerless motion capture gait kinematics. J Biomech. 2021;121: 110422. doi:10.1016/J.JBIOMECH.2021.110422

4. Wu G, Van Der Helm FCT, Veeger HEJ, Makhsous M, Van Roy P, Anglin C, et al. ISB recommendation on definitions of joint coordinate systems of various joints for the reporting of human joint motion--Part II: shoulder, elbow, wrist and hand. J Biomech. 2005;38: 981–992. doi:10.1016/J.JBIOMECH.2004.05.042

5. Kaneda JM, Seagers KA, Uhlrich SD, Kolesar JA, Thomas KA, Delp SL. Can static optimization detect changes in peak medial knee contact forces induced by gait modifications? J Biomech. 2023;152. doi:10.1016/J.JBIOMECH.2023.111569

6. Lin YC, Dorn TW, Schache AG, Pandy MG. Comparison of different methods for estimating muscle forces in human movement. Proc Inst Mech Eng Part H J Eng Med. 2012;226: 103–112. doi:10.1177/0954411911429401
